# Efficacy of electrical vestibular stimulation (VeNS) on adults with insomnia: A double-blind, randomized, sham-controlled trial

**DOI:** 10.1080/19585969.2025.2526547

**Published:** 2025-07-11

**Authors:** Teris Cheung, Joyce Yuen Ting Lam, Kwan Hin Fong, Calvin Pak-Wing Cheng, Yu-Tao Xiang, Tim Man Ho Li

**Affiliations:** a School of Nursing, The Hong Kong Polytechnic University, Hong Kong SAR, China; b The Mental Health Research Centre, The Hong Kong Polytechnic University, Hong Kong SAR, China; c Department of Psychiatry, The University of Hong Kong, Hong Kong SAR, China; d Department of Public Health and Medicinal Administration, Faculty of Health Sciences, University of Macau, Macau SAR, China; e Department of Psychiatry, The Chinese University of Hong Kong, Hong Kong SAR, China

**Keywords:** Vestibular stimulation, insomnia, randomised clinical trial, brain stimulation, efficacy

## Abstract

**Introduction:**

Insomnia, a widespread sleep disorder, affects a significant portion of the global population. This study is the first in Asia to evaluate the efficacy of electrical vestibular stimulation (VeNS) as a treatment for insomnia in Hong Kong adults, addressing a gap in non-pharmacological interventions.

**Methods:**

A double-blind, randomized, sham-controlled trial was conducted with 101 adults exhibiting insomnia symptoms. Participants were randomized into active VeNS or sham groups (1:1 ratio) and underwent twenty 30-minute VeNS sessions over four weeks. Psychological outcomes, including insomnia severity, sleep quality, and quality of life were assessed at baseline (T1), post-intervention (T2). Follow-up assessments were conducted at one- (T3) and three-month (T4) to evaluate the sustainability of VeNS effects.

**Results:**

Of 83 participants (40 VeNS and 43 sham-VeNS), the VeNS group showed significant reductions in insomnia severity at T2 (p = 0.03, d = -0.47) and T4 (p = 0.02, d = -0.32), alongside improved quality of life (i.e., role-physical) at T2.

**Conclusion:**

VeNS is a novel, non-invasive and safe neuromodulation device that may serve as an adjunct treatment for primary insomnia. The present findings provide a foundation for future multisite comparison studies to further evaluate VeNS efficacy.

**Trial registration:**

ClinicalTrials.gov Identifier: NCT04452981

## Introduction

Insomnia is a common psychiatric symptom affecting about one-third of adults in the UK, Hong Kong, and other regions (Wong and Fielding 2011; Morin and Jarrin 2022). The American Psychiatric Association (2022) reported that about 10% of people meet the criteria for an insomnia disorder, which includes challenges with starting or keeping sleep, as well as discontent with the amount or quality of sleep. Insomnia can be acute, intermittent, or chronic and is often comorbid with physical and mental health conditions (Sutton 2021), such as decreased quality of life (Lucena et al. 2020), hypertension (Johnson et al. 2019), impaired immune system functioning (Nieters et al. 2019), and cardiovascular disease (Larsson and Markus 2019). Cognitive behavioural therapy (CBT) is the preferred treatment due to its efficacy, safety, and durable benefit, but medications are widely used for insomnia symptoms (Sutton 2021).

While medications can effectively treat primary insomnia, many individuals hesitate to use them due to potential adverse effects, such as cognitive impairment and dependence (Glass et al. 2005; Gustavsen et al. 2008; Lader 2011; Lie et al. 2015; Krystal et al. 2019). Consequently, their extended application is not advocated (Glass et al. 2005; Schutte-Rodin et al. 2008; Qaseem et al. 2016; Riemann et al. 2017; Krystal et al. 2019). In contrast, CBT shows efficacy with minimal side effects (Glass et al. 2005; Gustavsen et al. 2008; Mitchell et al. 2012; Lie et al. 2015; Krystal et al. 2019; Morin et al. 2021), yet traditional Chinese medicine remains popular in Hong Kong, especially among younger generations (Liu et al. 2016). A recent pilot study suggested that integrated CBT (CBT-I) and acupressure could effectively treat insomnia (Ho et al. 2021). However, the effectiveness of CBT-I is often limited by the time, costs, and inadequate trained clinicians (Morin et al. 2021). Consequently, the majority of the individuals affected by sleeping disturbances in the community did not seek professional help in a timely fashion.

### Vestibular stimulation and insomnia

**Figure 1.**
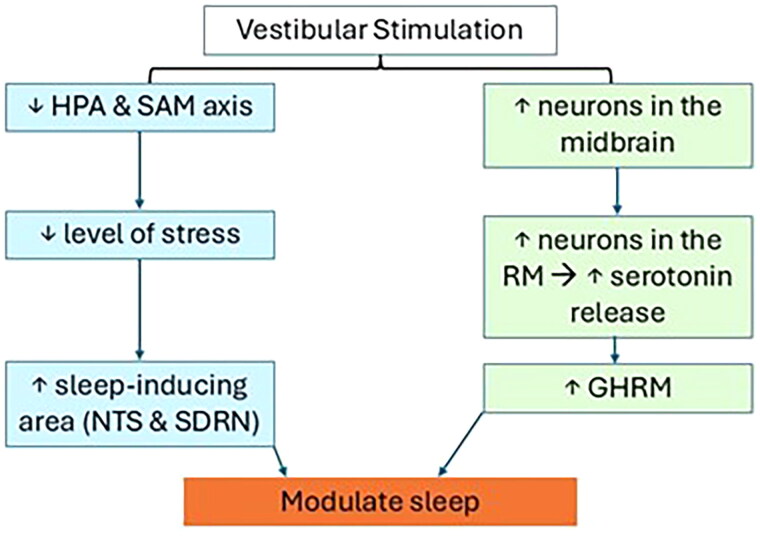
The mechanism of vestibular stimulation in modulating sleep. *Note.* In blue: Vestibular stimulation inhibits both the HPA axis and the sympathetic-adreno-medullary (SAM) axis, thereby decreasing stress (Sailesh and Mukkadan 2013a), and excites sleep-inducing areas such as the nucleus tractus solitarius (NTS) (Davis et al. 2004; Sailesh and Mukkadan 2013b) and the serotonergic dorsal raphe nucleus (Horowitz et al. 2005). In green: The activation of neurons in the midbrain periaqueductal gray matter excites neurons in the rostral medulla and increases serotonin release. Activation of serotonin receptors causes the secretion of growth hormone-releasing hormone (GHRH) (Conway et al. 1990), which promotes rapid eye movement (REM) and non-REM sleep in humans (Kerkhofs et al. 1993), thereby helping to modulate sleep (Snowball et al. 1997).

Vestibular stimulation has been considered one of the alternative treatment options for managing insomnia due to its effect in modulating sleep (see Figure 1).

There is strong physiological evidence demonstrating that the vestibular system can affect REM sleep (Morrison and Pompeiano 1970; Silvani and Dampney 2013; Besnard et al. 2018), and labyrinthine inputs influence the pontine reticular formation neurons involved in mediating the switching between sleep states (Yates 1998). The medial vestibular nucleus has projections in regions that mediate arousal, and some aspects of sleep receive orexinergic inputs from the lateral hypothalamus (Horowitz et al. 2005). The raphe nuclei, locus coeruleus, and reticular formation are central to the brain’s mechanisms for sleep regulation, receiving vestibular inputs critical for maintaining equilibrium and spatial orientation. The otolith organs, which are sensors of linear acceleration, project onto the pontine reticular formation, an area significantly involved in the modulation of sleep (Ebben 2024).

Evidence showed that electrical stimulation of the vestibular system can produce a rocking sensation that promotes sleep (Subramaniam et al. 2023), as physical rocking has been found to improve sleep in individuals with neuromuscular breathing problems (Iber et al. 1989), and the gentle swaying sensation produced by a vestibular device can significantly reduce sleep latency (Woodward et al. 1990). In other words, the input from the vestibular system to sleep-regulating nuclei could facilitate the synchronisation of the body’s internal state with tranquilising movements, promoting a state of relaxation and drowsiness conducive to sleep (Luxon and Pagarkar 2013).

### Objectives

The objectives of this study were to (1) evaluate the efficacy of VeNS on insomnia severity in community-dwelling adults in Hong Kong and (2) examine the association between VeNS data usage, insomnia severity, sleep quality, and quality of life. We hypothesised that participants in the VeNS group would experience a significant reduction in the insomnia severity compared to the sham group after four weeks, with effects maintained at 1- and 3-month follow-ups. In addition, we assumed that higher usage in the VeNS group would correlate with significant improvements in insomnia severity, sleep quality, and quality of life following the intervention.

## Methods

### Study design

The study protocol has been reported elsewhere (Cheung et al. 2023). This two-armed, double-blind, randomised, sham-controlled trial was conducted to evaluate the effects of a 4-week VeNS treatment on insomnia among adults in the general population of Hong Kong. The study strictly adhered to the Consolidated Standards of Reporting Trials (CONSORT) statement (Imran et al. 2021) and the ethical principles outlined in the Declaration of Helsinki (World Medical Association 2013).

### Subjects

#### Subject recruitment

Samples were recruited from our local universities and NGO in Hong Kong between 25 June - 19 November 2022. A flyer with a registration QR code was posted around the communal areas at the universities and NGO. Email invitations were sent to all staff, students and alumni across different faculties/departments. Project poster advertised in university’s Facebook and Twitter.

#### Eligibility criteria

Participants who met the eligibility criteria were included (Table 1).

**Table 1.** The eligibility criteria of participants.

Inclusion criteria	Exclusion criteria
ISI score ≥ 15 Ethnic Chinese, aged 18–60 years Ability to understand/read Chinese Not currently using prescribed or over-the-counter sleeping pills Capable of providing written informed consent Access to Wi-Fi and Bluetooth on iOS/Android mobile phones Ability to attend a face-to-face demonstration session and return-demonstrate use of the VeNS device Willingness to engage weekly with the project team *via* WhatsApp/telephone for compliance and technical issues No extreme lifestyle changes affecting sleep quality during the study Agreement not to use sleep trackers during the study No travel across time zones during the study	History of eczema, skin conditions, or inner ear diseases HIV/AIDS infection Use of beta-blockers, antidepressants, or other medications affecting neurostimulation History of stroke, epilepsy, severe head injury, or neurosurgery Active migraine with aura Significant communication impairments Metal implants in the brain or devices like pacemakers History of epilepsy Pregnant or breastfeeding women Cognitive impairments, including dementia and mild cognitive impairment History of major depressive disorder, psychotic disorder, bipolar disorder, or substance use disorders Regular use of antihistamines in the last 6 months History of malignancy in the past 12 months Diagnosis of myelofibrosis or myelodysplastic syndrome History of vestibular dysfunction or inner ear infections Previous use of any VeNS device

*Note.* VeNS = electrical vestibular stimulation

### Randomisation, allocation and masking

All eligible participants were fully informed about the randomisation procedures and were told that they had a 50% chance of receiving either the active VeNS or the sham VeNS in this trial. Each participant was assigned a unique identifier for randomisation. Randomisation was conducted by an independent statistician off-site using a stochastic minimisation program to balance the sex, age, and ISI scores of the participants. Block randomisation with blocks of 10 (total: 6 blocks) was used to allocate treatment groups. Participants from each block were randomly assigned to the active VeNS group or the sham VeNS group at a 1:1 ratio. To prevent information flow, both participants and research associates were blinded to group allocation to minimise potential contamination of the effects of VeNS or subject bias. The principal investigator was not involved in data collection. Participants were asked to guess their group allocation (VeNS vs. sham VeNS) during face-to-face follow-up meetings after completing the 4-week interventions to assess the success of subject blinding (Yeung et al. 2021).

### Intervention

**Figure 2.**
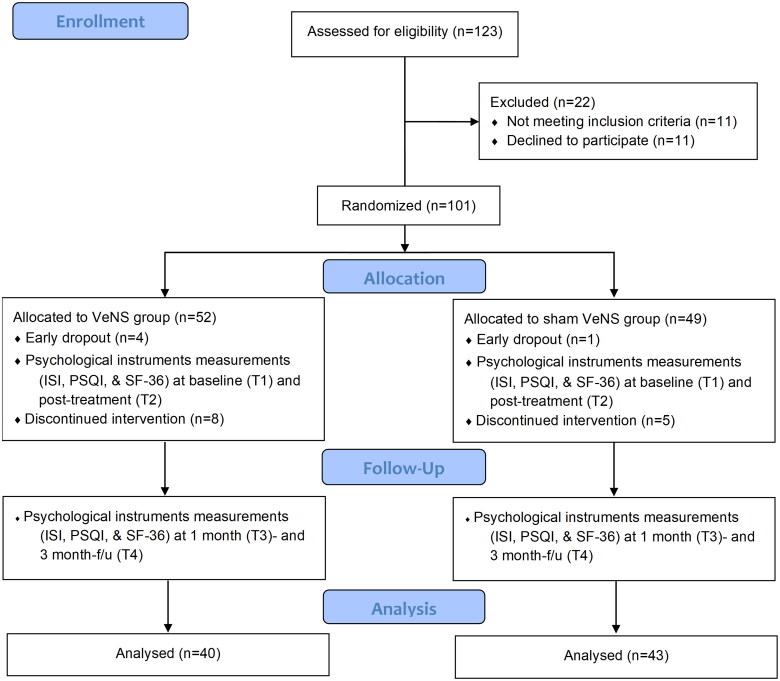
CONSORT flow diagram of subject recruitment and data collection at each time point. VeNS = Electrical Vestibular Stimulation; ISI = Insomnia Severity Index; PSQI = Pittsburgh Sleep Quality Index; SF-36 = 36-Item Short Form Health Survey

All participants in both the active and sham VeNS groups received a VeNS device from the Integrative Health Clinic, PolyU, following training provided by the research associates. Participants underwent 20 VeNS sessions at home, one hour prior to bedtime, with each session lasting 30 min over four weeks (i.e., Monday–Friday, total treatment time: 10 h). We believed this duration was sufficient to test VeNS efficacy on insomnia (Goothy and McKeown, 2020). Clinical assessments were conducted at baseline (T1), immediately post-intervention (T2), and at 1-month (T3) and 3-month follow-ups (T4) to evaluate immediate, short-term and long-term effects (Figure 2).

#### VeNS group

**Figure 3.**
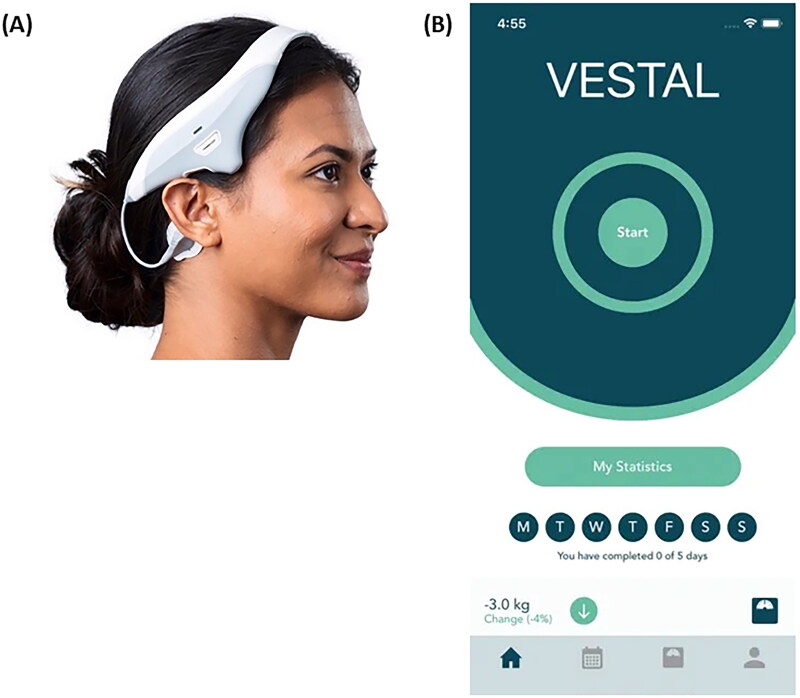
(A) The Modius Sleep device (Neurovalens^®^) as intended to be worn, with electrode pads over the mastoid processes. Identical appearance for Modius Sleep and sham devices. (B) The user interface of the study app (Vestal, Neurovalens^®^).

The VeNS device – namely Modius Sleep (MS) is a non-invasive, transdermal neurostimulation device that delivers low-level electrical currents to the individual’s head to treat insomnia. Participants placed the VeNS headset and applied two self-adhesive electrode pads on the mastoid processes behind the ears (Figure 3A). They remained in a sitting or resting position throughout the stimulation period. Next, they turned on the device and adjusted the stimulation level from 0–10 (max. 1 mA at 100 Hz) *via* a mobile app (Figure 3B) to achieve a tingling sensation. The stimulation frequency used in the present study based on a recent VeNS study (Goothy et al. 2022) in young adults, showing significant improvement on insomnia severity, emotional state and quality of life. Subjects initially felt a tingling sensation around the mastoid process until they felt a gentle swaying, indicating modulation of the vestibular nerve and indicating that subjects were reaching the optimal level of stimulation. After selecting the stimulation level, the VeNS device then delivered the stimulation when participants pressed the ‘start’ button, and the device automatically turned off after 30 min of stimulation. The device data, including total usage, average intensity, and average resistance, were collected *via* Bluetooth and stored on an encrypted server. The full intervention procedures and safety issues are described elsewhere (See Supplemental material).

#### Sham VeNS group

Participants in the sham-controlled group followed identical procedures as those in the VeNS group, but they received an initial stimulation for 30-second, followed by a decrease to 0 mA for 20-second. The sham group experienced a sham VeNS stimulation at a frequency of 0.8 Hz, which was chosen for its low sympathetic activation and minimal vestibular stimulation effects (Grewal et al. 2009; Macefield and James 2016). Previous study (Grewal et al. 2009) indicated that sinusoidal galvanic vestibular stimulation (GVS) delivered at 0.5–0.8 Hz could partially entrain muscle sympathetic nerve activity (MSNA), justifying the use of 0.8 Hz in this trial. Participants also experienced a sensation of skin tingling and vestibular stimulation, leading them to believe they had received active treatment. Due to user accommodation to the current, sensations of tingling or prickling typically subside after 30s (Nitsche et al. 2003; Paulus 2003). Participants are unable to distinguish between a device delivering 20 min of real stimulation and a sham device delivering only 30s of stimulation before switching off (Gandiga et al. 2006).

### Measures

The primary objective of this study was to evaluate the effects of VeNS on participants’ insomnia severity among adults in Hong Kong. Secondary objectives included examining the effects of VeNS data usage on sleep quality and quality of life.

#### Insomnia severity index (ISI)

Insomnia severity was assessed using the Chinese version of the ISI, which consists of seven items measuring day and night symptoms of insomnia in individuals. The ISI includes questions on perceived difficulty (Item 1), falling asleep (Item 2), time of awakening (Item 3), satisfaction with current sleep pattern (Item 4), interference with daily functioning (Item 5), noticeability of the impact of lack of sleep on others (Item 6), and the degree of perceived distress or concern caused by the sleep problem (Item 7). Subjects rated each question on a 5-point Likert scale (0-4), and the total score ranges from 0 to 28, with a score of 15-21 indicating moderately severe insomnia. The ISI has been used in the Chinese population with good psychometric properties, including a Cronbach’s alpha of 0.81 and item-to-total correlations in the range of 0.34-0.67 (Yu 2010).

#### Pittsburgh sleep quality index (PSQI)

The PSQI (Buysse et al. 1989) was used to assess sleep quality over the past month. It consists of 19 items: (1) the first 5 questions, which asked about their bedtime, time taken to fall asleep, wake-up time, actual sleep time, and time spent in bed during the past month; and (2) the remaining 14 questions, which asked about the frequency of sleep problems, use of sleeping pills to go to sleep, and daytime sleepiness on a Likert-type scale ranging from 0 to 3, with a higher value indicating more severe sleep problems. A score of > 5 indicates poor sleep quality, and the total score ranges from 0 to 21. An increase in the total score indicates a decrease in sleep quality. The Chinese version of the PSQI is a reliable and valid instrument for assessing sleep quality among the Hong Kong Chinese population (Ho et al. 2021).

#### 36-Item short form health survey (SF-36)

The Chinese version of the SF-36 (Li et al. 2002) was used to assess the quality of life (QoL) of participants. The SF-36 includes 36 questions related to an individual**’**s QoL in eight scales: physical functioning (PF), role-physical (RP), bodily pain (BP), general health perceptions (GH), vitality (VT), social functioning (SF), role-emotional (RE), and mental health (MH). The raw scores for each scale are transformed to a scale of 0-100, with higher scores indicating better QoL. The SF-36 is summarised in two component summary scores, the Physical Component Summary (PCS) and the Mental Component Summary (MCS). This Chinese version of the SF-36 is a valid and reliable instrument, with an overall Cronbach**’**s α coefficient of 0.943, while the Cronbach**’**s α coefficients for each of the dimensions were all > 0.70 (Liu et al. 2013).

### Statistical analyses

All statistical analyses were performed using R for Windows, v4.1.0. Descriptive analyses included means and standard deviations (SD) for continuous variables and numbers and percentages for categorical variables. Sociodemographic differences between the active VeNS group and the sham VeNS group were analysed using the Chi-square test and *t* test. Linear regression was employed to compare the improvement of the outcomes between the intervention group and the sham controls. Participants’ total usage time was also used to investigate the effectiveness of VeNS on insomnia, and total usage time was used as a continuous covariate in a linear regression model for the ISI total score. The linear regression analyses were adjusted for sociodemographic factors. Independent-samples *t* tests were used to test the baseline differences between the two groups. Bonferroni correction was used to adjust *p* values in multiple hypothesis tests (correlations of insomnia severity and sleep quality, and the main effects of the VeNS intervention between timepoints). Missing data among the study participants were imputed using a random forest imputation. Analysis was on an intention-to-treat basis. Effect sizes for each outcome were indexed by Cohen’s *d*. A *p*-value < 0.05 was considered to indicate statistical significance.

## Results

### Study sample

A total of 83 individuals (age = 40.8 ± 12.25 years; age ranged 21-60) were screened and recruited for the study. This number exceeded our estimated sample size, as evidenced by the fact that our Block 1 participants already had 30% attrition in both groups in the second week of VeNS intervention due to inpatient admission, sickness and technical phobia. Thus, the project team decided to invite more eligible participants to participate in this study. All eligible eighty-three individuals were randomly assigned to the intervention group (*n* = 40) and the sham-controlled group (*n* = 43). There was no difference in usage time between the active VeNS group (usage = 642.7 ± 72.54 min) and the sham VeNS group (usage = 637.7 ± 60.95 min). There were no statistically significant differences in sociodemographic variables between the two groups. More females (71%) than males (29%) participated in the study. Most of the participants lived with parents/children (67%), had completed tertiary education (55%), and had family incomes ranging from HK$20,000 to HK$29,999 (52%). There were four participants who were divorced or widowed, while the others were either single or married. A total of 71% of participants did not have a diagnosis of insomnia. Most of them (69%) did not use medication. There were no statistically significant differences between the two groups in terms of primary and secondary outcomes at baseline (see Table 2).

**Table 2.** Sociodemographic and baseline measurements between the intervention group and the sham group (*N* = 83).

	Intervention (*n* = 40)	Sham (*n* = 43)	*p*
Age	39.6 (12.06)	41.8 (12.48)	0.42
Total usage (mins)	642.7 (72.54)	637.7 (60.95)	0.86
Sex			>.99
Male	12 (30)	12 (28)	
Female	28 (70)	31 (72)	
Marital Status			0.34
Single	23 (57)	17 (40)	
Married	16 (40)	23 (53)	
Divorced	1 (2)	2 (5)	
Widowed	0 (0)	1 (2)	
Living Status			0.75
Alone	7 (18)	7 (16)	
Parents/Children	28 (70)	28 (65)	
Others	5 (12)	8 (19)	
Education			0.76
Primary	0 (0)	1 (2)	
Secondary	14 (35)	15 (35)	
Post-Secondary	4 (10)	3 (7)	
Bachelor or above	22 (55)	24 (56)	
Monthly Family Income (HK$)			0.63
$20,000 - $29,999	24 (60)	19 (44)	
$30,000 - $39,999	5 (12)	5 (12)	
$40,000 - $49,999	4 (10)	6 (14)	
$50,000 - $59,999	2 (5)	4 (9)	
$60,000 - $79,999	4 (10)	5 (12)	
$80,000 or above	1 (2)	4 (9)	
Occupation			0.49
Housewife/Houseman	6 (15)	12 (28)	
Clerk/Admin	14 (35)	8 (19)	
Professionals	5 (12)	5 (12)	
Technical Staff	5 (12)	6 (14)	
Unemployed	8 (20)	11 (26)	
Retired	2 (5)	1 (2)	
Diagnosis (insomnia)			0.35
Yes	14 (35)	10 (23)	
No	26 (65)	33 (77)	
Nonprescribed medication to aid sleep			>.99
Yes	13 (32)	13 (30)	
No	27 (68)	30 (70)	
ISI	20.0 (4.18)	18.7 (4.60)	0.18
Hour in bed	7.7 (1.54)	7.7 (1.69)	0.91
SF-36			
PF	76.8 (21.90)	78.3 (21.67)	0.76
RP	34.0 (39.94)	44.5 (41.65)	0.25
RE	21.4 (37.06)	27.6 (37.93)	0.46
VT	30.5 (19.26)	35.5 (25.44)	0.33
MH	41.8 (20.33)	46.5 (24.62)	0.36
SF	47.1 (22.66)	52.4 (30.26)	0.38
BP	56.9 (24.31)	58.7 (24.84)	0.75
GH	34.1 (23.02)	36.7 (21.14)	0.60
PSQI-total	13.0 (4.03)	12.7 (3.31)	0.75

*Note.* HKD$1 is approximate to USD$7.8. ISI = Insomnia Severity Index; SF-36 = 36-Item Short Form Health Survey, includes PF, physical functioning; RP, role–physical; RE, role–emotional; VT, vitality; MH, mental health; SF, social functioning; BP, bodily pain; GH, general health; PSQI = Pittsburgh Sleep Quality Index

### Effect of the intervention

Tables 3 and 4 show the associations of insomnia severity and sleep quality at baseline and posttest in the two groups. Significant positive correlations were found between the above variables in the VeNS group (*r* =.42-.62, *p* < .01) and in the sham VeNS group (*r* =.51-.69, *p* < .01).

**Table 3.** Pearson correlational matrix of primary (insomnia severity) and secondary outcome (sleep quality) measures (*N* = 83).

		Mean	SD	1	2	3	4
1	pre-ISI	19.19	4.42	--			
2	post-ISI	14.49	5.25	.529**	--		
3	pre-PSQI	12.80	3.60	.534**	.209	--	
4	post-PSQI	9.92	4.06	.348**	.618**	.519**	--

Note. ** p<.01. pre = baseline; post = posttest; ISI = Insomnia Severity Index; PSQI = Pittsburgh Sleep Quality Index

**Table 4.** Pearson correlational matrix of insomnia severity and the secondary outcome (sleep quality) between groups.

Group			Mean	SD	1	2	3	4
VeNS (*n* = 40)	1	pre-ISI	19.98	4.14	--			
	2	post-ISI	14.15	3.82	.620**	--		
	3	pre-PSQI	12.98	3.98	.424**	.010	--	
	4	post-PSQI	9.50	3.55	.436**	.476**	.574**	--
Sham VeNS (*n* = 43)	1	pre-ISI	18.47	4.61	--			
	2	post-ISI	14.81	6.32	.528**	--		
	3	pre-PSQI	12.63	3.25	.659**	.374*	--	
	4	post-PSQI	10.30	4.50	.331*	.685**	.508**	--

Note. **p < .01, *p < .05. pre- = baseline; post- = posttest; ISI = Insomnia Severity Index; PSQI = Pittsburgh Sleep Quality Index

Table 5 reveals the effect of the intervention. Compared with the control group, the VeNS group demonstrated significant improvement in insomnia severity (ISI) at T2 (*p* = 0.03, *d* = -0.47) and T4 (*p* = 0.02, *d* = -0.32). Participants’ total usage time was not found to be associated with the effect of VeNS on insomnia. For secondary outcomes, the VeNS group had better improvement in physical functioning compared to the controls. VeNS had an immediate effect on role limitations due to physical health (SF-36-RP), as shown in the improvement of the intervention at T2 (*d* = 0.26), whereas the improvement was not sustained at T3 and T4. The effect on the secondary outcome was considered small (see Supplementary Materials).

**Table 5.** Comparison of the outcome measures before and after the intervention and follow-up periods.

	Δ T2 - T1 (Intervention vs. Sham controlled)					Δ T3 - T1 (Intervention vs. Sham controlled)					Δ T4 - T1 (Intervention vs. Sham controlled)				
	Mean (SD)		*β* (95%CI)	*p*	*d*	Mean (SD)		*β* (95%CI)	*p*	*d*	Mean (SD)		*β* (95%CI)	*p*	*d*
ISI	−5.88 (3.68)	−3.72 (5.31)	2.28 (0.22, 4.33)	0.03*	−0.47	−6.21 (5.34)	−4.46 (4.17)	2.19 (−0.12, 4.50)	0.06	−0.32	−6.38 (4.77)	−4.46 (4.72)	2.62 (0.40, 4.84)	0.02*	−0.32
Hour in bed	−0.26 (1.70)	0.56 (1.62)	0.67 (−0.08, 1.42)	0.08	−0.63	0.37 (3.00)	0.98 (2.98)	0.50 (−0.87, 1.87)	0.47	−0.29	0.39 (3.49)	0.22 (2.27)	−0.49 (−1.92, 0.93)	0.49	0.03
SF-36-PF	3.52 (22.89)	7.23 (16.31)	4.24 (−5.48, 13.97)	0.39	−0.43	6.52 (18.98)	5.92 (17.38)	0.13 (−8.60, 8.86)	0.98	−0.14	2.62 (27.38)	4.58 (22.34)	1.20 (−10.54, 12.95)	0.84	−0.18
SF-36-RP	17.16 (34.61)	6.12 (42.52)	−18.79 (−34.71, −2.86)	0.02*	0.26	12.97 (52.47)	17.86 (40.84)	−4.23 (−23.72, 15.26)	0.67	−0.16	7.05 (46.49)	8.83 (37.44)	−4.93 (−23.62, 13.75)	0.60	−0.09
SF-36-RE	19.14 (40.02)	16.62 (44.20)	−4.40 (−23.54, 14.74)	0.65	0.12	15.16 (46.98)	23.62 (34.82)	1.86 (−16.67, 20.39)	0.84	−0.20	16.39 (40.59)	16.18 (35.58)	−4.89 (−22.00, 12.21)	0.57	0.02
SF-36-VT	10.41 (15.84)	6.94 (19.45)	−0.19 (−8.40, 8.03)	0.96	0.24	9.00 (20.95)	6.26 (18.52)	−4.18 (−13.49, 5.13)	0.37	0.20	7.18 (22.63)	5.94 (17.14)	−2.54 (−11.98, 6.90)	0.59	0.11
SF-36-MH	11.41 (17.18)	5.53 (17.71)	−5.06 (−13.14, 3.01)	0.22	0.42	6.51 (20.20)	4.95 (19.83)	−2.78 (−11.74, 6.17)	0.54	0.13	5.84 (18.87)	3.36 (18.04)	−4.80 (−13.17, 3.57)	0.26	0.21
SF-36-SF	14.57 (19.94)	10.32 (19.70)	−4.93 (−13.82, 3.97)	0.27	0.16	9.80 (20.81)	7.68 (18.46)	−3.57 (−12.44, 5.30)	0.42	0.06	11.81 (23.78)	6.30 (19.80)	−8.13 (−17.36, 1.10)	0.08	0.26
SF-36-BP	4.27 (20.53)	10.92 (20.94)	6.76 (−3.09, 16.60)	0.18	−0.38	−0.06 (23.65)	3.68 (20.47)	4.81 (−5.29, 14.91)	0.35	−0.15	0.77 (27.40)	4.38 (19.13)	2.33 (−8.96, 13.63)	0.68	−0.13
SF-36-GH	6.49 (16.08)	6.35 (16.18)	1.23 (−6.20, 8.66)	0.74	−0.03	2.77 (18.67)	5.24 (16.11)	3.46 (−4.63, 11.54)	0.40	−0.18	3.54 (16.98)	1.80 (14.62)	−1.39 (−8.32, 5.55)	0.69	0.10
PSQI-total	−3.47 (3.57)	−2.51 (4.00)	0.65 (−1.05, 2.35)	0.45	−0.31	−2.78 (4.35)	−1.73 (3.50)	0.92 (−0.94, 2.77)	0.33	−0.30	−2.97 (3.87)	−2.43 (3.98)	0.81 (−1.05, 2.66)	0.39	−0.14

*Note.* * *p* < .05. T1 = baseline; T2 = posttest; T3 = 1-month follow-up; T4 = 3-month follow-up; ISI = Insomnia Severity Index; SF-36 = 36-Item Short Form Health Survey; includes PF, physical functioning; RP, role–physical; RE, role–emotional; VT, vitality; MH, mental health; SF, social functioning; BP, bodily pain; GH, general health; PSQI = Pittsburgh Sleep Quality Index

## Discussion

This is the first study evaluating the efficacy of VeNS in adults with insomnia symptoms in Asia. Our findings suggest that VeNS is effective in reducing insomnia severity and improving participants’ physical well-being immediately after the 4-week intervention and at the 3-month follow-up, compared to the sham VeNS group. However, the effect size is considered small (Cohen’s *d* = 0.21-0.32). Interestingly, VeNS appeared to be less effective for all primary and secondary outcome measures (reduction of insomnia, improvement of sleep quality, and quality of life) at the 1-month follow-up (all *P*s > 0.05) (see Table 5). It is apparent that participants in both groups share similarities, as insomnia severity is significantly and positively correlated with sleep quality after VeNS stimulation (see Table 4). In addition, it is noteworthy that 25% of participants in the VeNS group believed they were allocated to the sham group, while 40% of the sham group thought they were in the VeNS group. This relatively high percentage of incorrect guesses in the blinding process has likely biased our findings and indicates a strong placebo effect in the sham group. Our speculation was affirmed by Um et al. (2022) that conducted an experimental study using transcutaneous trigeminal electrical modulation (TTEN) on 13 patients aged 19-64 with insomnia. They assessed changes in insomnia using the ISI, PSQI, Epworth Sleepiness Scale (ESS), and polysomnography. Participants were administered 20-min TTEN sessions for four weeks. Their findings reported a statistically significant reduction in all subjective measures (ISI, PSQI, and ESS), but no significant differences in objective polysomnography parameters pre- and postintervention at the 1-month follow-up (*p* = 0.913). Another experimental study (Goothy and McKeown 2020) used electrical VeNS on 20 participants who received 30-min stimulation one hour before sleep for two consecutive weeks. The results showed a significant decrease in ISI scores post-stimulation (*p* < 0.000). Nonetheless, both studies (Goothy and McKeown 2020; Um et al. 2022) were limited by small sample sizes, large age variations, lack of a sham-controlled group, and failure to perform sample size calculations, which may have biased their results.

An open-label trial was conducted by Marshall et al. (2010) on 105 adult (age 21-65) participants who received 1 h of VeNS daily for 30 days to evaluate the changes in insomnia severity. Pre- and post-ISI scores showed a statistically significant improvement in the reduction of insomnia severity [mean (SD) 17.8 (4.0) and 11.8 (5.4), *p* < .001], and researchers concluded that vestibular nerve stimulation was a therapeutic intervention for chronic insomnia, with significant improvement in ISI mean scores and classifications, sleep onset latency, total sleep time, wake after sleep onset and sleep efficiency. Of particular note is that this trial is also limited by the lack of a control group and the absence of objective sleep data derived by actigraphy or polysomnography. Thus, the study findings were entirely reliant on subjective self-reported evaluations.

An older multisite, doubled-blind, randomised sham-controlled trial (Krystal et al. 2010) was conducted on 198 healthy normal sleepers in six sites in the United States that also used VeNS over the mastoid process. Of these, 101 participants received VeNS stimulation, and 97 were randomised to sham VeNS. There were no significant differences between treatment groups in self-reported global difficulty falling asleep; however, there was a trend for subjects treated with vestibular stimulation to report shorter sleep onset latency on the treatment night (mean = 44.2, SD = 43.7) vs. sham-treated subjects (mean = 60.7, SD = 65.9) with *F* = 3.3, *p* < 0.08. Their findings have suggested that the vestibular system and sleep are closely linked, and the rocking sensation produced during the electrical stimulation of the vestibular system, specifically, the mastoid process, may promote sleep. Physiological evidence has consistently shown that the vestibular system can affect rapid eye movement (REM) sleep (Morrison and Pompeiano 1970; Hobson et al. 1998; Datta et al. 1999; Eisensehr et al. 2001), that there is an influence of labyrinthine inputs on the pontine reticular formation neurons involved in mediating switching between sleep states (Horowitz et al. 2005) and that the medial vestibular nucleus has projections to regions mediating arousal and some aspects of sleep that receive orexinergic inputs from the lateral hypothalamus (Horowitz et al. 2005).

Findings from the aforementioned studies have clearly indicated that interventional studies should not simply rely on self-reported subjective data, which may induce social desirability bias. Future replication of similar studies should incorporate sleep trackers and sleep journals to triangulate objective and scientific study findings.

Hyperarousal (increased excitability) has long been a major culprit for inducing insomnia symptoms, evidenced by increased high-frequency electroencephalography (EEG) and sympathetic system activation in individuals with insomnia (Bonnet and Arand 2010; Levenson et al. 2015). Central dysregulation of the noradrenergic system is a major cause of hyperarousal and subsequent anxiety and insomnia (Yamamoto and Shinba 2009). In other words, insomnia is closely linked to hyperarousal and anxiety. We speculate that once participants in the VeNS group completed the 4-week intervention and returned the device to the project team, it might trigger immediate acute anxiety, which may induce self-perceived insomnia. This possibly explains why VeNS seemed to have no significant effect on insomnia symptoms, sleep quality and quality of life at the 1-month follow-up, but the effect on ISI and quality of life became significant at the 3-month follow-up. Most importantly, vestibular stimulation promotes sleep by regulating growth hormone and thyroid hormones and hence reducing stress (Sailesh & Muikkadan, 2013b). Thus, it seems reasonable for participants not to report any significant changes at the 1-month follow-up, and they started to subjectively feel the improvement in insomnia severity at the 3-month follow-up. It is somewhat interesting to note that VeNS did not lead to any significant changes in sleep quality evaluated by the PSQI at the three measurement timepoints (immediately after 4 weeks of stimulation and at the 1-month and 3-month follow-ups) (all *P*s > 0.05). These findings could be explained by our relatively small sample size, which limits the power of the study, and more importantly, our correlational matrix identified a significant correlation between insomnia severity and sleep quality (Tables 3 and 4) at baseline and poststimulation for both groups. Future replications of similar studies should include larger-scale multisite RCTs with sham-controlled groups to minimise potential placebo effects (Jiang et al. 2019).

Our findings have identified that VeNS stimulation may lead to some improvements in quality of life, particularly role limitations due to physical health (RP). Other domains (i.e., PF, RE, VT, MH, SF, BP, and GH) were not found to be statistically significant. These findings contradict those of a cross-sectional study (Zhang et al. 2017) involving 297 adolescents and 318 parents, with 80 and 93 individuals with insomnia, respectively, that aimed to explore the relationship between insomnia and quality of life in China. Insomnia was evaluated by the ISI, whereas quality of life was evaluated by health-related quality of life (HRQoL). The results showed that participants with insomnia had a significantly lower HRQoL than those without insomnia. Although Zhang et al.’s study identified the association between insomnia and quality of life, the causal relationship and long-term impact of insomnia on quality of life remain unknown. Nevertheless, our study findings have confirmed Zhang et al.’s findings that insomnia is linked with quality of life to a certain extent.

Using sham VeNS that delivers a subtherapeutic dose is a well-known hurdle in neurostimulation studies to avoid the likelihood of a high attrition rate (Starling et al. 2018). In this study, we included a sham control group; however, sham subjects also received VeNS stimulation for 30 s in each session throughout the intervention. This short duration may potentially give sham-group subjects a strong perception that they are receiving the active VeNS treatment. More importantly, we did not assess vestibular sensational differences between the VeNS group and the sham VeNS group that could potentially confound our results (Barclay and Barclay 2014; McClure et al. 2015; Mischoulon et al. 2015; Wagenseil et al. 2018). Because our study samples are sourced from a community-dwelling general population instead of a homogenous sample, it is difficult to closely monitor device use compliance on a daily basis throughout the intervention period. Some participants may fail to strictly comply with the four-week VeNS stimulation due to sickness, technical phobia, fear of adverse effects, etc., which may affect the measurement outcomes.

In addition, we found that the usage data of VeNS was not significantly correlated with insomnia severity. In other words, the null hypothesis of our second hypothesis was established. This can partially be explained by the fact that the VeNS devices in the VeNS group power-off and lock after 30 min of use for the purposes of research safety and adherence. Participants cannot extend the use of VeNS to ‘speedily’ improve insomnia or sleep quality, and this hurdle may deter continuous use and good compliance in the use of the VeNS device throughout the four-week intervention period.

Neuroimaging evidence points to the role of hyperarousal in the pathophysiology of insomnia with inconsistent findings (Drake et al. 2014; Dodds et al. 2017). Existing neuromodulation research has used different stimulation protocols, such as transcranial direct current stimulation (tDCS) and repetitive transcranial magnetic stimulation (rTMS). Nonetheless, there is significant variability in the stimulation parameters, study samples, and sleep outcomes (Provencher et al. 2019). In our investigation, we found no discernible gender disparities in the outcomes of the VeNS intervention. This contrasts with the findings of Zhang et al. (2023), who reported gender-based differences in tasks that are vestibular-perturbed or demanding, attributable to anatomical variations in the vestibular system. Nevertheless, a comparable randomised, controlled clinical trial that employed VeNS on individuals with Parkinson’s Disease experiencing sleep disturbances also showed no significant gender-based differences post-intervention (Goothy et al. 2022).

In summary, existing neuromodulation studies for insomnia are scant. There is a pressing need for more robust randomised controlled trials to establish international standardised protocols for VeNS to reduce insomnia and promote better quality of life in the near future.

### Limitations

Although VeNS is effective in easing insomnia symptoms immediately poststimulation and at 3 months, there are some limitations that should be addressed. First, we did not obtain each participant’s VeNS stimulation level in the VeNS group; hence, we cannot conclude which level is optimal for this study. Second, participants were sourced from community-dwelling adults, and study findings may not be generalisable to other age groups, for example, adolescents and older adults over age 65 or above. Third, we were unable to confirm whether participants were experiencing chronic insomnia. Chronic insomnia is defined as having symptoms at least three times per week for a duration of three months or longer (Robbins and Quan 2024). The two self-reported measures used in this study, the ISI and PSQI, assessed sleep conditions over a period of two weeks to one month prior, which may not adequately capture chronic insomnia. Future studies should incorporate diagnostic tools or longitudinal assessments to evaluate different insomnia conditions. Fourth, the present study reports findings from Hong Kong only, which may not be generalisable to other cultural contexts in Asian or European regions.

## Conclusion

VeNS is a novel, non-invasive and safe device designed to reduce insomnia severity and improve sleep quality (both physical and emotional aspects) immediately at post-stimulation and at the 3-month follow-up. In view of the increased prevalence of insomnia in Hong Kong, incorporating the VeNS device as an adjunct treatment option alongside pharmacological treatments and psychotherapy could help reduce the global disease burden and improve overall mental wellbeing for Hong Kong citizens.

## Supplementary Material

Supplementary Materials_v2.docx

## Data Availability

The original contributions presented in the study are included in the article/supplementary material, and further inquiries can be directed to the corresponding author.
