# Supplementary material for: Efficacy of electrical vestibular stimulation (VeNS) on adults with insomnia: A double-blind, randomized, sham-controlled trial
Source: Dialogues Clin Neurosci. 2025 Jul 11;27(1):236–48. doi: 10.1080/19585969.2025.2526547 (PMC12258234; doi:10.1080/19585969.2025.2526547)
Supplement: Supplementary Materials_v2.docx [file TDCN_A_2526547_SM2910.docx]

**Supplementary Materials**

**Figure S1.** **Changes in primary and secondary outcomes between the VeNS and sham VeNS groups.**


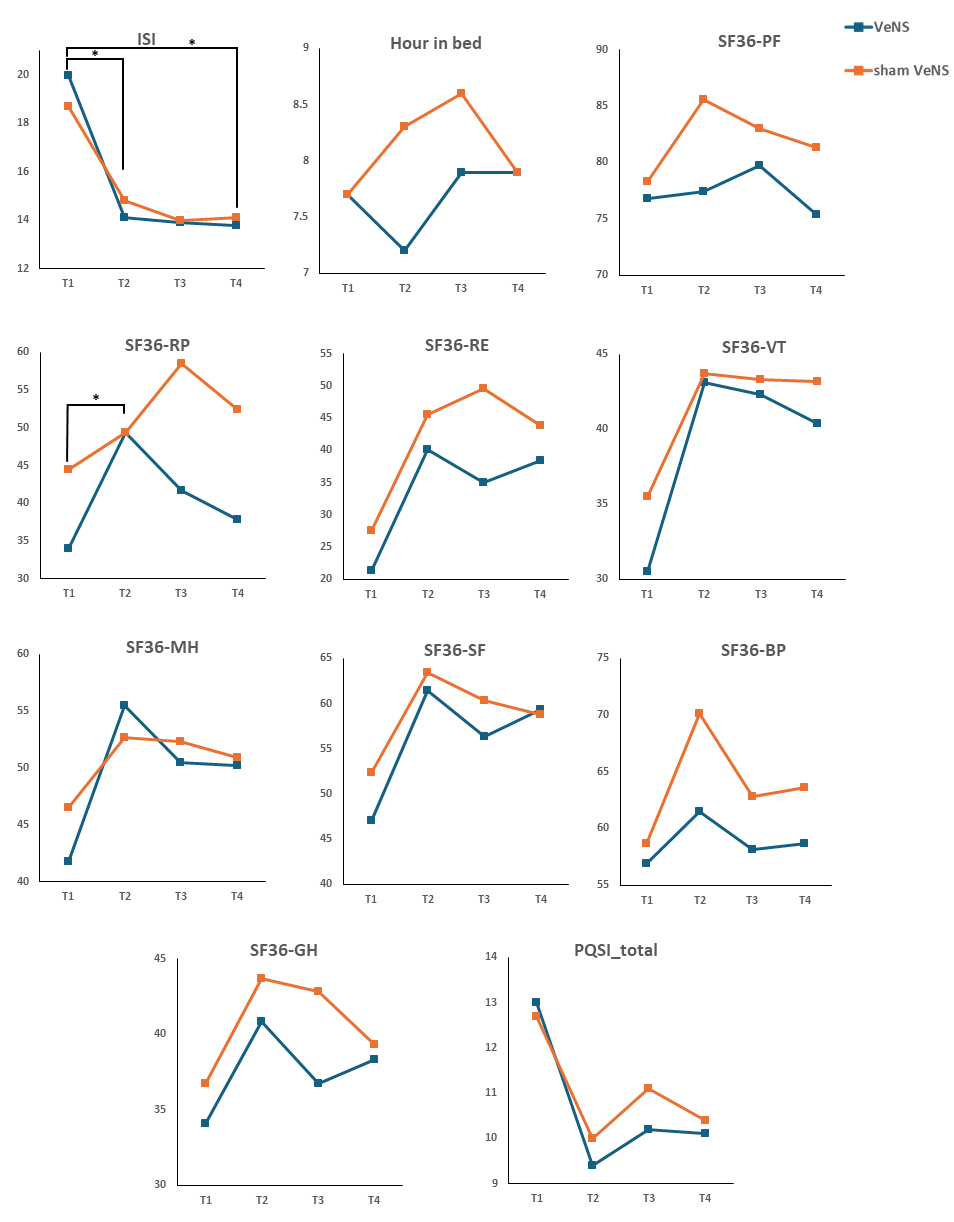


*Note.* *indicates p < 0.05. Abbreviations: VeNS = Electrical Vestibular Stimulation; T1 = baseline; T2 = posttest; T3 = 1-month follow-up; T4 = 3-month follow-up; ISI = Insomnia Severity Index; SF-36 = 36-Item Short Form Health Survey; includes PF, physical functioning; RP, role–physical; RE, role–emotional; VT, vitality; MH, mental health; SF, social functioning; BP, bodily pain; GH, general health; PSQI = Pittsburgh Sleep Quality IndexISI = Insomnia Severity Index; PSQI = Pittsburgh Sleep Quality Index; SF-36 = 36-Item Short Form Health Survey
